# Endoplasmic Reticulum Stress in Intestinal Epithelial Cell Function and Inflammatory Bowel Disease

**DOI:** 10.1155/2015/328791

**Published:** 2015-02-10

**Authors:** Katherine Luo, Stewart Siyan Cao

**Affiliations:** Columbia University College of Physicians and Surgeons, New York, NY 10032, USA

## Abstract

In eukaryotic cells, perturbation of protein folding homeostasis in the endoplasmic reticulum (ER) causes accumulation of unfolded and misfolded proteins in the ER lumen, which activates intracellular signaling pathways termed the unfolded protein response (UPR). Recent studies have linked ER stress and the UPR to inflammatory bowel disease (IBD). The microenvironment of the ER is affected by a myriad of intestinal luminal molecules, implicating ER stress and the UPR in proper maintenance of intestinal homeostasis. Several intestinal cell populations, including Paneth and goblet cells, require robust ER function for protein folding, maturation, and secretion. Prolonged ER stress and impaired UPR signaling may cause IBD through: (1) induction of intestinal epithelial cell apoptosis, (2) disruption of mucosal barrier function, and (3) induction of the proinflammatory response in the gut. Based on our increased understanding of ER stress in IBD, new pharmacological approaches can be developed to improve intestinal homeostasis by targeting ER protein-folding in the intestinal epithelial cells (IECs).

## 1. Endoplasmic Reticulum Stress and the Unfolded Protein Response

In eukaryotic cells, the endoplasmic reticulum (ER) is a membrane-bound organelle crucial for the folding of secretory and membrane proteins, lipid biosynthesis, and regulation of intracellular Ca^2+^ and oxidation-reduction signaling. ER protein folding and modification are highly sensitive to disturbances of ER homeostasis, including altered glycosylation, oxidative stress, energy deprivation, ER Ca^2+^ depletion, increased mRNA translation, altered metabolic status, and inflammatory stimuli. The accumulation of unfolded and misfolded proteins in the ER lumen, termed ER stress, activates the unfolded protein response (UPR), which resolves the protein folding defect and restores ER homeostasis. In mammalian cells, three protein sensors on the ER membrane initiate the UPR: inositol-requiring kinase 1*α* (IRE1*α*), pancreatic ER eIF2*α* kinase (PERK), and activating transcription factor 6*α* (ATF6*α*). In the absence of ER stress, ER chaperone binding immunoglobulin protein (BiP), also known as glucose-regulated protein 78 (GRP78), binds to the luminal domains of the ER stress sensors, thereby maintaining their inactive states. Accumulation of unfolded/misfolded proteins during ER stress dissociates BiP from the luminal domains of the three stress sensors, thereby activating them and initiating UPR signaling (Hetz et al. [1, 2]).

IRE1 is the most conserved ER stress transducer among the three protein sensors.* IRE1α* and* IRE1β* are two* IRE1 *genes that have been identified in mice and humans. Expression of* IRE1α* is ubiquitous, while* IRE1β* has primarily been identified in epithelial cells of the gut and respiratory tract. IRE1*α* is a type I transmembrane protein with an endoribonuclease domain and a serine/threonine kinase domain in its cytosolic portion. Once BiP has been dissociated from IRE1*α*, the luminal domain of IRE1*α* undergoes homooligomerization and* trans*-autophosphorylation in order to activate its endoribonuclease and kinase activities. Activated IRE1*α* splices a 26-base intron from an mRNA encoding a transcription factor called X-box-binding protein 1 (XBP1). The resulting translational frame-shift-containing mRNA produces a functionally active XBP1s isoform, which is a potent CREB/ATF basic leucine zipper (bZIP) transcription factor. XBP1s orchestrates UPR signaling by inducing the expression of genes which involve ER protein folding, secretion, phospholipid biosynthesis, ER expansion, and ER-associated protein degradation (ERAD). The IRE1*α*-XBP1 pathway is required for murine embryonic development as well as the differentiation, survival, and function of various secretory cells such as plasma cells, pancreatic *β* cells and acinar cells, and hepatocytes. Surprisingly, genetic or pharmacological inhibition of IRE1*α* can also improve the stress response in certain cells. Several molecular pathways have been shown to contribute to the deleterious effects of IRE1*α*. The kinase domain of IRE1*α* binds with TNF*α* receptor-associated factor 2 (TRAF2) in the cytoplasm. Phosphorylation of TRAF2 leads to subsequent activation of the NF-*κ*B and c-Jun N-terminal kinase (JNK) pathways, which then contribute to inflammatory and proapoptotic signaling in the cell [1–3]. Additionally, IRE1*α* binds to proapoptotic proteins Bax and Bak on the mitochondrial outer membrane and promotes mitochondrion-dependent cell death. Thirdly, in the presence of cellular stress, the endoribonuclease domain of IRE1*α* targets a subset of ER-localized mRNAs, a process called regulated IRE1-dependent decay of mRNA (RIDD). Recent studies showed that RIDD exacerbates cell death upon prolonged/high ER stress by degrading mRNAs encoding prosurvival proteins [1, 4].

PERK is another ER stress sensor and type I transmembrane protein with a serine/threonine kinase domain on its cytosolic portion. In response to ER stress, PERK becomes activated in a manner similar to that of IRE1—that is, by dissociating from BiP and undergoing homooligomerization and* trans*-autophosphorylation. Activated PERK suppresses global protein synthesis and alleviates the ER protein-folding load by phosphorylating Ser51 on the *α* subunit of eukaryotic translation initiation factor 2 (eIF2*α*), which impedes translation initiation. Three cytosolic kinases have been discovered in mammals to phosphorylate eIF2*α* at Ser51, one of which is the double-stranded RNA-activated protein kinase (PKR). Phosphorylated eIF2*α*, despite effectively inhibiting global protein synthesis, selectively promotes translation of a subset of mRNAs, most notably an mRNA encoding a bZIP transcription factor called ATF4. ATF4 plays an important role in a number of stress-response pathways by inducing expression of UPR-associated transcription factors, ER chaperones, intracellular trafficking machinery, and regulators of autophagy and antioxidative responses. CCAAT/enhancer-binding protein homologous protein (CHOP) is a downstream, apoptosis-promoting target of ATF4. CHOP-mediated apoptosis has been linked to a number of ER stress signaling pathways. In response to ER stress, ER oxidase 1*α* (ERO1*α*) transcriptional expression is enhanced by CHOP activity, potentially contributing to oxidative damage by generating reactive oxygen species (ROS) in the ER. ERO1*α* also stimulates inositol-1,4,5-trisphosphate receptor on the ER membrane and, therefore, induces Ca^2+^ release from the ER. Increased cytosolic Ca^2+^ activates Ca^2+^/calmodulin-dependent protein kinase II and leads to apoptosis [1, 2, 4]. In addition, CHOP inhibits prosurvival protein Bcl-2 and induces proapoptotic factors Bim, telomere repeat binding factor 3 (TRB3), and death receptor 5 (DR5). CHOP activity also directly leads to the accumulation of DR5 protein, which undergoes ligand-independent activation and promotes apoptosis via the caspase-8 pathway during prolonged ER stress [5]. CHOP has recently been shown to interact with ATF4 to induce genes encoding protein synthesis machinery, which contributes to oxidative stress, energy depletion, and apoptotic cell death in ER-stressed cells [6].

ATF6*α* is a type II transmembrane protein with a CREB/ATF bZIP domain at its N-terminal cytoplasmic portion. ATF6*α* is part of the family of regulated intramembrane proteolysis- (RIP-) regulated bZIP transcription factors. In response to ER stress, ATF6*α* dissociates from BiP and travels to the Golgi apparatus, where it is cleaved first in its luminal domain by site-1-protease (S1P) and then in the transmembrane region by S2P. A freed cytosolic fragment p50 of ATF6*α* is released and migrates into the nucleus. ATF6*α* p50 then transactivates ER chaperone genes (e.g.,* Grp78*,* Grp94*, and* P58* ^*IPK*^), the components of ER-associated protein degradation (ERAD), and induces ER biogenesis. In cells with ER stress, ATF6*α* promotes protein folding, maturation, and secretion. The absence of ATF6*α* can result in cell death due to increased susceptibility to prolonged ER stress. In addition to ATF6*α*, several RIP-regulated bZIP transcription factors, including OASIS/CREB3L1, CREBH, and Luman, have been identified. ER stress activates these transcription factors which play diverse and crucial roles in various cell types [2, 3].

## 2. ER Stress and the UPR in Intestinal Epithelial Cells

The mammalian digestive tract encounters trillions of microbes and various food metabolites, exposing it to numerous antigens. Exquisite interactions between the gut microbiome and peripheral tissues, and the immune system are integral in regulating homeostasis in the intestine. Both innate and adaptive immune cell populations modulate the balance between homeostasis and inflammation in the gastrointestinal tract. Increasing evidence suggests that the epithelial cells lining the bowel wall not only function as a barrier but also actively participate in the maintenance of mucosal homeostasis. Four secretory cell types are found in the intestinal epithelia of mice and humans: Paneth, goblet, enteroendocrine, and absorptive cells, all of which are differentiated from a common and constantly renewing Lgr5^+^ intestinal stem cell (ISC) population [7, 8]. Another population of stem cells, called +4 population, can also differentiate* in vivo* and* in vitro* into all four IEC lines. In contrast to Lgr5^+^ ISC, this population is relatively quiescent and is radiation-resistant [9].

Paneth cells are critical in the innate immune and system for warding off bacteria, fungi, and certain viruses. Histologically, they are pyramid-like columnar epithelial cells that reside at the bottom of small intestine crypts [10]. Cell-autonomous MyD88-dependent toll-like receptors (TLRs) allow Paneth cells to directly sense intestinal bacterial cells and byproducts. Activation of the TLRs triggers expression and secretion of multiple antimicrobial factors, including lysozyme, cryptdins/*α*-defensins, and phospholipase A2 [11]. Paneth cells may also limit infiltration of the intestinal barrier by both commensal and pathogenic microbes, allowing for maintenance of intestinal homeostasis and equilibrium among different populations of gut microflora. Crohn's ileitis has been linked to Paneth cell dysfunction, such as reduced secretion of antimicrobial peptides [12].

Unlike Paneth cells, goblet cells produce large amounts of gel-forming and cell-surface mucins in both the small and large intestines under normal physiological conditions. The major component of the mucus layer of the gut is MUC2 mucin, which, prior to secretion into the intestinal lumen, is intracellularly processed via N-glycosylation in the ER and O-glycosylation in the Golgi apparatus [13]. The presence of mucin stops infiltration by bacteria, fungi, viruses, and associated toxic byproducts. Intestinal homeostasis is safeguarded by its complex mucus layer. Spontaneous colitis and colitis-associated colon cancer resulted from* Muc2* knock-out in mice [14]. Moreover, goblet cell deficiency and/or dysfunction are hallmarks of ulcerative colitis (UC) [15]. Nevertheless, the physiological relevance of goblet cell pathology is not yet well understood in the context of UC and Crohn's disease (CD).

Protein misfolding in the ER of IECs has increasingly been suggested to directly contribute to IBD. IBD patients with active disease typically have increased markers of ER stress in ileal and/or colonic epithelia [16–19]. However, even unaffected mucosal tissues of IBD patients have signs of impaired integrated stress response (i.e., decreased levels of eIF2*α* phosphorylation) to situations such as ER stress, oxidative stress, viral infection, inflammation, amino acid deficiency, and heme depletion [20, 21]. Increased accumulation of MUC2 precursor in the ER of goblet cells, reduced mucin secretion, and impaired mucus layers were observed in mice expressing a mutant* Muc2* gene. Strikingly, activation of innate and adaptive immunity with an induced Th17 response in the colon was also exhibited by mutant* Muc2* mice,a process that is similar to that of human UC [18, 22]. Accumulation of misfolded MUC2 precursors in the ER of colonic goblet cells in some UC patients also suggested that the protein folding defect is physiologically relevant to goblet cell pathology in the pathogenesis of UC [18]. IL10, an anti-inflammatory cytokine essential for intestinal homeostasis, was found to alleviate ER stress and promote goblet cell secretion of mucin [23]. Similarly, mutant MUC2 mucin folding and secretion in murine colon was able to be bolstered by administration of glucocorticoids. The glucocorticoids induced ER chaperones and ERAD components in goblet cells and, therefore, mitigated intestinal inflammation.

Recently, a number of UPR regulators have been linked to the pathogenesis of IBD [24]. UPR gene* XBP1* has been associated with IBD by human genetic studies [25]. Paneth cell ablation was generated by induction of ER stress via IEC-specific deletion of the gene encoding XBP1 in animal studies.* Xbp1* conditional knockout mice also displayed goblet cell deficiency and mucin secretion dysfunction, along with spontaneous inflammation in small intestine and impaired host defense to enterobacterial infection [16]. Knockout of* Xbp1* alleles stimulated JNK and NF-*κ*B pathways and production of inflammatory mediators in the mucosa via massive activation of IRE1*α* in the ileal epithelium. Further studies showed that Paneth cell-specific ablation (*Defa6-Cre*) of* Xbp1* led to a similar phenotype of spontaneous enteritis as that observed in mice with total IEC deletion (*Villin-Cre*) of* Xbp1*, implying that Paneth cell-specific UPR plays an essential role in mucosal homeostasis in murine ileum [26]. The mammalian GI tract specifically expresses IRE1*β*, an IRE1 isoform, which conferred protective effects against dextran sodium sulfate- (DSS-) induced colitis in mice [27, 28]. IRE1*β* also degrades* Muc2* mRNA in goblet cells, allowing for optimized folding and secretion of MUC2 mucin. In the absence of* IRE1β* expression, MUC2 precursors accumulated in the ER lumen of colonic goblet cells, which presented with induction of ER stress and distended ER [29]. However, the mechanism of IRE1*β* regulation of goblet cell function and mucosal homeostasis during colitis and infection in the gut is still elusive.

A recent study suggested that XBP1 knockout-induced autophagy is correlated with activation of ER stress markers phosphorylated PERK/eIF2*α* and ATF4. Deletion of both* Xbp1 *and autophagy gene* Atg1611* in the IECs of mice led to spontaneous transmural inflammation of the ileum, the same phenotype as Crohn's ileitis in humans. IRE1*α*, NF-*κ*B, TNF*α* signaling, and apoptosis of ileal IECs were also observed to be activated in the* Xbp1* ^−/−^ */Atg1611* ^−/−^ mice [26]. XBP1 and autophagy may play partially compensatory roles to suppress proinflammatory signaling and maintain mucosal homeostasis and, therefore, support Paneth cell function in mice.

Polymorphisms of* Nod2* have also been shown in several GWAS papers to be associated with CD in populations of Puerto Rican descent [30].* Nod2* is expressed in a variety of cells, including macrophages and dendritic cells, and at low levels in IECs. It encodes a bacteria-sensing protein that recognizes D-glutamyl-*meso*-diaminopimelic acid, a peptidoglycan found primarily in Gram-negative bacteria. However, Nod2 has also been shown to be involved in the autophagy pathway through association with* Atg16l1*. One study showed that injection of* Nod2* agonists into the peritoneal cavities of thioglycollate-primed mice induced markers of autophagy, suggesting that* Nod2* could initiate autophagy. Moreover, NOD2 and ATG16L1 both colocalized at bacterial entry sites, and the* Nod2* polymorphism L1007insC, which is strongly associated with CD, failed to induce bacterial autophagy as ATG16L1 did not localize to the plasma membrane [31]. The PERK-eIF2*α*-ATF4 branch of the UPR also plays an important role in IEC function and intestinal homeostasis. Marked decreases in UPR-associated transcription factors and ER chaperones and ileal IEC autophagic activation were observed in mice with IEC-specific expression of nonphosphorylatable eIF2*α* (*AA* ^*IEC*^). Under electron microscopy,* AA* ^*IEC*^ Paneth cells exhibited impaired production of secretory proteins and granules as well as fragmented ER and degenerated mitochondria. Components of ER protein translocation, including signal peptidase complex catalytic subunit 11c, signal sequence receptor 1, and translocation protein Sec63, were compromised, while* Lysozyme* and* Cryptdin* mRNAs translation were attenuated [32]. Moreover,* AA* ^*IEC*^ mice displayed increased susceptibility to ileal* Salmonella* infection, though they failed to develop spontaneous enterocolitis as seen in* Xbp1* ^Δ*IEC*^ mice. These findings suggest that eIF2*α* phosphorylation-dependent UPR signaling promotes ER protein translocation, protein folding in the ER, and autophagy and, as a result, controls Paneth cell function [33]. Both* AA* ^*IEC*^ mice and mice with a deficiency of eIF2*α* kinase PKR in nonhematopoietic cells were more susceptible to DSS-induced colitis in the colon [33–35]. In contrast, ablation of* Chop*, the gene encoding a key regulator of apoptosis in ER-stressed cells, alleviated DSS-induced colitis in mice [36]. Because a whole-body deletion of* Chop* was used in that study, it is not clear whether this phenotype is due to the loss of CHOP in epithelial or hematopoietic cells.

RIP-regulated bZIP transcription factors regulate multiple aspects of intestinal epithelial function. DSS-induced colitis increased in severity in mice containing a hypomorphic mutation of the gene encoding S1P in mice [37]. Given that S1P targets several ER stress-induced bZIP transcription factors (e.g., ATF6*α*, Luman, OASIS, CREBH, and SREBPs), it is possible that more than one of these transcription factors play a role in IEC function and mucosal homeostasis [2, 3]. Recently, it was found that* Oasis* ^−/−^ mice possessed colonic goblet cells containing abnormal ER and mucous vesicles, supporting the hypothesis that OASIS controlled terminal differentiation of goblet cells [38]. Upon DSS challenge,* Oasis* ^−/−^ mice showed increasingly severe signs of colitis with elevated ER stress and apoptotic markers in IECs [39]. A murine model with nonhematopoietic-specific deletion of* Atf6α* revealed that ATF6*α* induced ER chaperone genes in colonic epithelial cells during inflammation, thereby mitigating DSS-induced colitis. Furthermore, mice lacking* P58* ^*IPK*^ (a gene encoding an important ER cochaperone) displayed hyperactive inflammation in the colon with induction of markers of ER stress and apoptosis following DSS treatment, supporting the role of the ER chaperone response as a protective response against DSS-induced colitis [40]. AGR2 is a protein disulfide isomerase localized in the ER and assists with protein folding. ER stress in the intestinal epithelium, disruption of Paneth cell homeostasis, and CD-like granulomatous ileocolitis were associated with deletion of* Agr2* [41]. In another study, normal maturation and secretion of MUC2 mucin in murine colonic goblet cells required AGR2 activity [42].

## 3. ER Stress and UPR in IBD Therapeutics

Development of therapies for IBD is faced with extraordinary challenges due to our poor understanding of the disease. Current immunosuppressive medications involve substantial risks and side effects [43]. Potential therapies may target ER stress, as ER homeostasis plays a critical role in IEC function. Chemical chaperones tauroursodeoxycholate (TUDCA) and 4-phenylburyrate (PBA) are two FDA-approved compounds found to reduce ER stress in cultured IECs that were treated with inflammatory stimuli. Moreover, feeding either TUDCA or PBA to DSS-induced,* Il10* ^−/−^ and *Tnf* ^Δ*ARE*^ murine models of IBD reduced intestinal inflammation by alleviating ER stress in the IECs [40]. However, it remains unclear how other cell types, such as immune cells and fibroblasts, are affected by chemical chaperones in response to inflammatory insults. In the* ApoE* ^−/−^ mouse model of atherosclerosis, PBA treatment induced IL10, IL35, and Foxp3 expression and elevated the T regulatory cell (Tregs) count, which assisted in dampening chronic inflammation of the arterial wall [44]. Normal physiological mucosal homeostasis required the presence and activity of CD4^+^Foxp3^+^ Tregs and cytokines IL10 and IL35 [45, 46], suggesting the importance of understanding how differentiation, activation, and migration of Tregs and other immune cells in IBD models are affected by PBA. Glutamine is an important fuel for rapidly-dividing cells including IECs. 2,4,6-Trinitrobenzenesulfonic acid- (TNBS-) induced colitis in rats was ameliorated by glutamine administration through reduction of ER stress, oxidative stress, colonic epithelial cell apoptosis, and the inflammatory response. Similarly, chemically induced ER stress in cultured Caco-2 cells was also suppressed by glutamine [47]. It is still unknown whether glutamine directly or indirectly alleviates ER stress in IECs (e.g., directly by assisting in protein folding or indirectly by affecting energy homeostasis and mitochondrial function). Salubrinal is a small molecule inhibitor of eIF2*α* dephosphorylation and exerts its effects by inhibiting eIF2*α* phosphatase GADD34 [48]. In a new murine model with a double deficiency in IL10 and NADPH oxidase 1, salubrinal was able to mitigate the spontaneous UC-like phenotype [49]. In a recent study, salubrinal was shown to exhibit similar protective function in mice with DSS-induced colitis, probably by boosting adaptive UPR signaling BiP and ATF4 and heat-shock protein 70 [50]. However, the role of eIF2*α* phosphorylation in restoring mucosal homeostasis in the gut via non-IEC machineries remains poorly understood.

## 4. Discussion

Inflammatory bowel disease is the 2nd most common inflammatory disease in the US [51]. Despite years of intense research, the etiology of this debilitating disease remains elusive. There is currently no cure for IBD, which commonly requires a lifetime of care for patients. In the gut of mammals, the interface between the exogenous molecules, microflora, and the immune system is maintained by the intestinal epithelial layer. IBD may be caused by failure of IECs to modulate inflammatory responses, impairing mucosal homeostasis. Cells with high burdens of protein folding and secretion are significantly more vulnerable to changes in ER homeostasis, which can potentially induce inflammatory gene expression [52, 53]. Biosynthesis, maturation, and secretion of antimicrobial peptides and mucins can be stimulated by a variety of environmental signals such as bacterial molecules, bile salts, cholinergic stimuli, host innate and adaptive immune mediators, and some cellular stresses through pathways including NF-*κ*B and MAPK signaling [54–57]. The protein secretory capacities of Paneth and goblet cells may be overwhelmed by augmented demand of production of antimicrobial peptides and mucins. Meanwhile, inflammatory and oxidative insults may generate ER stress through perturbation of ER homeostasis through poorly understood mechanisms [52]. Exposure to high levels of exogenous antigens, cytokines/chemokines, toxins, and reactive oxidative/nitrosative species in the intestinal lumen places a large demand on the protein processing facilities of IECs. As a result, IECs also may require robust UPR signaling to survive the challenging environment and fulfill their duties. In contrast, the function and homeostasis of IECs may be impaired by chronic ER stress and defective UPR, potentially causing epithelial cell death, compromising barrier function, and segueing into intestinal inflammation. ER stress induction in the intestinal epithelia of IBD patients, the various UPR genes associated with IBD, and evidence from animal studies all suggest that a perturbed ER homeostasis may contribute to the pathogenesis of IBD.
